# Complete genome sequence of the *Bacillus paralicheniformis* strain HAS-1

**DOI:** 10.1128/mra.00337-24

**Published:** 2024-07-05

**Authors:** Lorna Catalina Can-Ubando, Ninfa Ramírez-Durán, Elisabet Aranda, Gauddy Lizeth Manzanares-Leal, Ayixon Sánchez-Reyes, Gabriela Ángeles de Paz, Keila Isaac-Olivé, Horacio Sandoval-Trujillo

**Affiliations:** 1 Laboratorio de Investigación en Microbiología Médica y Ambiental, Facultad de Medicina, Universidad Autónoma del Estado de México, Toluca, México, México; 2 Instituto Universitario de Investigación del Agua, Universidad de Granada, Granada, Spain; 3 Investigador por México, CONAHCYT-Instituto de Biotecnología, Universidad Nacional Autónoma de México, Cuernavaca, Morelos, Mexico; 4 Laboratorio de Investigación en Teranóstica, Facultad de Medicina, Universidad Autónoma del Estado de México, Toluca, Mexico; 5 Departamento de Sistemas Biológicos, Laboratorio de Producción de Biológicos, Universidad Autónoma Metropolitana-Xochimilco, Ciudad de México, Mexico; University of California Riverside, Riverside, California, USA

**Keywords:** complete genome, *Bacillus paralicheniformis*, strain HAS-1, halotolerant

## Abstract

We present the complete genome of the halotolerant strain *Bacillus paralicheniformis* HAS-1.

## ANNOUNCEMENT

*Bacillus paralicheniformis* (gram-positive bacillus), endospore-forming bacterium, was described in 2015 (1). It is potentially controller of phytopathogenic nematodes (2), polystyrene degrader (3), and plant activity growth promoter (4). We present the complete genome of strain HAS-1, isolated from a hypersaline sediment (0.1 mg/mL) from Texcoco’s Lake, Mexico (19°28*'*42*''*N, 98°58*'*26*''*W), in June 2019, identified as *Bacillus paralicheniformis*.

HAS-1 was inoculated using Czapek agar medium (37°C/7–10 days) and cultured on moderately halophilic bacteria medium (5). It is halotolerant with NaCl growth range 0%–10% (optimum, 0%) and a pH range of 7–12 (optimum, 8).

The Promega Wizard Genomic Kit-(A1125) was used for DNA extraction, according to manufacturer’s instructions. Gene 16S rRNA was amplified (primers 27F 5′-AGA GTT TGA TCM TGG CTC AG-3′ and 1492R 5′-TAC GGT TAC CTT GTT ACG ACT T-3′) by PCR (6) and sequenced by Psomagen. The consensus sequence (1,436 bp) was obtained using the Bioedit software (v.7.09) (7). BLAST software (8) and EzBiocloud database (9) were used for sequence comparison. Identity percentages were found with *Bacillus haynesii* (97.63%, BLAST, accession number PP438760.1) and *Bacillus paralicheniformis* (98.52%, EzBiocloud, accession number KY694465).

To sequence the HAS-1 genome, a single colony was streaked on nutrient broth agar and incubated at 37°C (72 hours). The cell biomass grown was collected aseptically for DNA purification. High-molecular-weight (HMW) DNA was isolated using the Quick-DNA HMW MagBead Kit (Zymo, D6060), according to manufacturer’s instructions. Long-read sequencing was performed using the MinION platform at the Massive Sequencing and Bioinformatics Core located at IBT-UNAM.

We conducted library preparation and sequencing on the MinION platform using unsheared genomic DNA (1 µg) with the Ligation Sequencing Kit SQK-LSK109. For multiplexing, we employed the EXP-NBD104 barcoding kit, and sequencing was carried out on the R9.4.1 flow cell. We performed base calling and reads demultiplexing using Guppy-v4.4.1 in high-accuracy model (10). The raw-reads quality assessment with NanoPlot showed 82,233 reads with a mean length and an N50 value of 3047.6 and 12,599 nucleotides, respectively. Read quality was filtered using NanoFilt version-2.8.0 (11), and adapters were trimmed with Porechop-v0.2.4 (12).

More than 99% of reads were above quality cutoffs >Q7: 82,211 reads with mean read quality of 12.4. Filtered reads were assembled using minimap2 miniasm pipeline-v0.3 (13) and polished with racon and minipolish (14). Finally, the genome was rotated to start at DNA nucleotide sequence to the first position with rotate scripts-v1.0 (15). The genome was annotated with the NCBI Prokaryotic Genome Annotation Pipeline (v 6.1) (16). Default parameters were used for all software unless otherwise noted. The annotation reveals that HAS-1 has genes that could encode enzymes involved in the metabolism of aromatic compounds, as diclofenac and ketoprofen.

A chromosome-level assembly was achieved, resulting in one single replicon with a size of 4,527,785 bp, 45.50% G + C, and an average reads sequencing coverage depth of 40×. The completeness and contamination were estimated with CheckM-v1.2.2 (17) on 98.82% and 0.06%, respectively. The average nucleotide identity (ANI) was estimated according to NCBI workflow (18). The genome of HAS-1 shows an ANI of 99.06% against *B. paralicheniformis*-type assembly: GCA_001042485.2.

A total of 4,640 genes were annotated with 4,436 protein-coding genes, 2 genes correspond to non-coding RNA, 24 rRNAs (8 5S, 8 16S, and 8 23S), and 81 tRNAs.

## Data Availability

The consensus sequence of the 16S rRNA gene was deposited in GenBank under accession number PP438760. This genome is deposited at NCBI with the accession number CP082896; the version described in this article is the version CP082896.2. The biosample number associated is SAMN20057200 and bioproject number PRJNA743662. Raw reads were deposited into SRA with the accession SRR27946199.
